# Iron Intake and Dietary Sources in the Spanish Population: Findings from the ANIBES Study

**DOI:** 10.3390/nu9030203

**Published:** 2017-02-27

**Authors:** Mᵃ de Lourdes Samaniego-Vaesken, Teresa Partearroyo, Josune Olza, Javier Aranceta-Bartrina, Ángel Gil, Marcela González-Gross, Rosa M. Ortega, Lluis Serra-Majem, Gregorio Varela-Moreiras

**Affiliations:** 1 Department of Pharmaceutical and Health Sciences, Faculty of Pharmacy, CEU San Pablo University, Boadilla del Monte, 28668 Madrid, Spain; l.samaniego@ceu.es (M.d.L.S.-V.); t.partearroyo@ceu.es (T.P.); 2 Department of Biochemistry and Molecular Biology II and Institute of Nutrition and Food Sciences, University of Granada, 18100 Granada, Spain; jolza@ugr.es (J.O.); agil@ugr.es (Á.G.); marcela.gonzalez.gross@upm.es (M.G.-G.); lluis.serra@ulpgc.es (L.S.-M.); 3 Department of Preventive Medicine and Public Health, University of Navarra, 31008 Pamplona, Spain; jaranceta@unav.es; 4 CIBEROBN, Biomedical Research Networking Center for Physiopathology of Obesity and Nutrition, Carlos III Health Institute, 28029 Madrid, Spain; 5 ImFINE Research Group, Department of Health and Human Performance, Technical University of Madrid, 28040 Madrid, Spain; 6 Department of Nutrition, Faculty of Pharmacy, Madrid Complutense University, 28040 Madrid, Spain; rortega@ucm.es; 7 Research Institute of Biomedical and Health Sciences, University of Las Palmas de Gran Canaria, Las Palmas de Gran Canaria, 35016 Las Palmas, Spain; 8 Spanish Nutrition Foundation (FEN), 28010 Madrid, Spain

**Keywords:** micronutrients, iron, ANIBES Study, dietary survey, food sources

## Abstract

Background: Iron deficiency is one of the most common nutritional problems in the world. It is frequent in both developed and developing countries and mainly affects women of childbearing age and children. Methods: Results were derived from the ANIBES cross-sectional study using a nationally-representative sample of the Spanish population (9–75 years, *n* = 2009). A three-day dietary record, collected by means of a tablet device, was used to obtain information about food and beverage consumption and leftovers. Results: Total median dietary iron intake was 9.8 mg/day for women and 11.3 mg/day for men. Highest intakes were observed among plausible adolescent reporters (13.3 mg/day), followed by adults (13.0 mg/day), elderly (12.7 mg/day), and children (12.2 mg/day). Prevalence of adequacy for iron intakes as assessed by EFSA criteria was higher than for the Spanish Recommended Iron Intake values in all age groups. Females had lower adequacy than males for both criteria, 27.3% and 17.0% vs. 77.2% and 57.0% respectively. Cereals or grains (26.7%–27.4%), meats and derivatives (19.8%–22.7%), and vegetables (10.3%–12.4%) were the major iron contributors. Conclusion: Higher iron intakes were observed in adolescents and were highest for non-heme iron. The prevalence of adequate iron intake according to EFSA criteria was higher than compared to national recommendations, and women had the lowest intakes. Therefore, there is a need to define standard dietary reference intake to determine inadequate iron intakes in the Spanish population.

## 1. Introduction

Iron is an essential nutrient of public health relevance required for many metabolic processes in the human body. It is part of haemoglobin and therefore crucial for the delivery of oxygen to the cells. It is also a structural component of many enzymes needed for a wide range of processes, such as phagocyte antimicrobial activity, neurotransmitter synthesis and function, and the production of DNA, collagen, and bile acids [1]. Iron deficiency is the most common and widespread nutritional disorder in the world [2,3]. As well as affecting a large number of children and women in non-industrialized countries, it is the only nutrient deficiency that is also significantly prevalent in virtually all industrialized nations. There are no current global figures for iron deficiency, but using iron deficiency anaemia as an indirect indicator, it can be estimated that most preschool children and pregnant women in non-industrialized countries, and at least 30%–40% in industrialized countries, are iron deficient [2]. Women, especially adolescents consuming low-energy diets, are at high risk of iron deficiency: anaemia is three times more frequent in adolescent girls who have tried to lose weight during the preceding 12 months compared with those who have not [3]. According to the World Health Organization (WHO), in the Spanish population anaemia prevalence ranges from 14% to 18% in children and in women of reproductive age, respectively [4]. Román-Viñas et al. observed a prevalence of iron intake inadequacy of 10%–21% of the Estimated Average Requirement (EAR) in a number of European populations when analyzing the European Nutrition and Health Report [5]. 

Considerable amounts of iron must be provided by the diet to replace the iron that is lost from the body (through blood loss and exfoliation of skin and gastrointestinal cells) and growth requirements [1]. Many dietary factors can hamper or promote absorption of this mineral, but the most important determinant of dietary iron absorption is systemic iron need: more is absorbed in a state of iron deficiency and less is absorbed when iron depots are replete [6]. In circumstances of marked iron requirements, however, the influence of dietary factors on iron absorption may become limiting. There are three main dietary factors related to iron status: quantity of iron, quality of iron, and the composition of diet [7]. 

Dietary iron is present in different forms and varying concentrations in a broad range of foods. There are two kinds of iron in the usual diet with respect to mechanisms of absorption: haem and non-haem iron. The richest sources of iron are cereals, vegetables, nuts, eggs, fish, and meat. Iron is also added to food as a fortificant in many countries [8,9] and is available as supplements [10]. Haem iron is 2–6 times more bioavailable from the diet than non-haem iron [11]. Daily Recommended Iron Intakes (DRI) for women of childbearing age is 18 mg/day and 8 mg/day for men and postmenopausal women [12]. Estimated Average Requirement (EAR) for iron is 8.1 mg/day for fertile women, 6 mg/day for men, and 5 mg/day for postmenopausal women [13]. 

Among the most remarkable limitations of dietary surveys in Spain are the use of non-harmonized methods for collecting intake data and the important occurrence of potential misreporting [9]. Assessing the validity of self-reporting among study participants might increase overall accuracy and representativeness of results. In addition, the use of new methods for collecting dietary information is necessary: completing food records is time-consuming hard work and can therefore be very inconvenient. Methods using information and communication technologies may also improve quality and accuracy [14]. 

There is a need for better and updated knowledge of micronutrient intakes in the Spanish population to prevent and/or delay adverse effects resulting from inadequate intakes at different stages of life. Dietary intake assessment of major iron sources is relevant because of the high anaemia prevalence amongst women of childbearing age. The ANIBES study is one of the few national dietary surveys in Spain to have collected comprehensive data with novel collection methods. Moreover, is the only representative one of the Spanish population using new technologies such as tablet devices to record food intakes and leftovers. The present study focuses on evaluating iron dietary intakes in the Spanish population according to age, gender and considering misreporting, but also to examine the main food sources that contribute to the mineral dietary intake. 

## 2. Materials and Methods

The complete design, protocol and methodology of the ANIBES study have been described in detail elsewhere [15,16,17].

### 2.1. Sample

**Figure 1:**
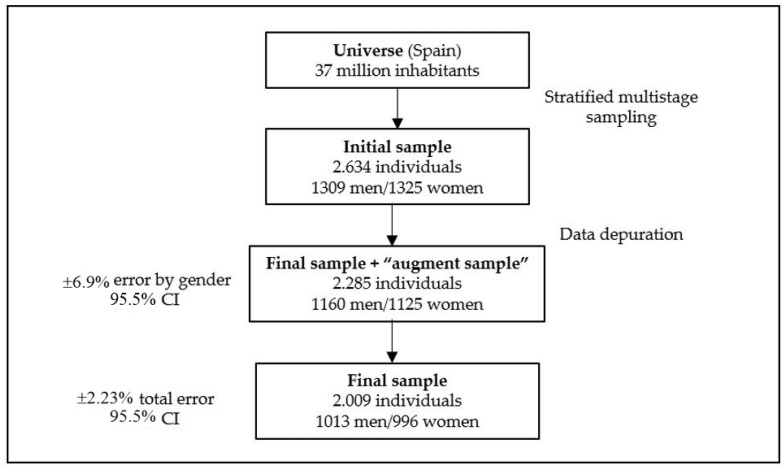
Sample collection design for the ANIBES study.

The ANIBES study is a cross-sectional study conducted using stratified multistage sampling. To guarantee better coverage and representativeness, the fieldwork was performed at 128 sampling points across Spain. The design of the ANIBES study aims to define a sample size that is representative of all individuals living in Spain, aged 9–75 years, and living in municipalities of at least 2000 inhabitants. The initial potential sample consisted of 2634 individuals, and the final sample comprised 2009 individuals (1013 men, 50.4%; 996 women, 49.6%). In addition, for the youngest age groups (9–12, 13–17, and 18–24 years), an “augment sample” was included to provide at least *n* = 200 per age group (error ± 6.9%) (Figure 1). The augment sample is the process of increasing the amount of interviews for a particular subgroup within the population in order to achieve an adequate number of interviews to allow analysis of population subgroups or segments that wouldn’t normally yield a sufficient number of interviews in a main random survey, without the expense of increasing the sample size for the whole survey. Therefore, the random sample plus augment sample comprised 2285 participants. The sample quotas according to the following variables were age groups (9–12, 13–17, 18–64, and 65–75 years), gender (men/women), and geographical distribution (Northeast, East, Southwest, North–Central, Barcelona, Madrid, and Balearic and Canary Islands) and locality size: 2000 to 30,000 inhabitants (rural population); 30,000 to 200,000 inhabitants (semi-urban population), and over 200,000 inhabitants (urban population). The following exclusions were applied: people that lived in colleges, hospitals, and other institutions; those strictly following a diet for medical tests or to determined pathologies; those who had an acute disease (common cold, gastroenteritis, chickenpox, etc.); and those working in fields related to market research, advertising, or journalism. In addition, people not strictly following a medical diet, those following recommendations for diabetes, hypertension, hypercholesterolemia, hypertriglyceridemia or hyperuricemia, women who were pregnant or at lactation period, those with allergies or intolerances, and those with metabolic diseases such as hyperthyroidism or hypothyroidism were included. Finally, other factors for sample adjustment were considered: unemployment rate, percentage of foreigners (immigrant population), physical activity level assessed by The International Physical Activity Questionnaire (IPAQ) [18], tobacco use, and education or economic level. The fieldwork for the ANIBES study was conducted from mid-September 2013 to mid-November 2013, and two previous pilot studies were also performed. To equally represent all days of the week, study subjects participated during two weekdays and one weekend day. The final protocol was approved by the Ethical Committee for Clinical Research of the Region of Madrid, Spain [16].

### 2.2. Food and Beverage Records

Study participants were provided with a tablet device (Samsung Galaxy Tab 2 7.0, Samsung Electronics, Suwon, Korea) and trained in recording information by taking photos of all food and drinks consumed during the three days of the study, both at home and outside. Photos had to be taken before beginning to eat and drink, and again after finishing, so as to record the actual intake. Additionally, a brief description of meals, recipes, brands, and other information was recorded using the tablet. Participants who declared or demonstrated that they were unable to use the tablet device were offered other options, such as using a digital camera and paper record and/or telephone interviews. A total 79% of the sample used a tablet, 12% a digital camera, and 9% opted for a telephone interview. As no differences in the percentage of misreporting were found according to the type of device used to assess dietary intake, we used the measurements of the three assessment methods in the analysis. In addition to details of what and how much was eaten, for each eating/drinking event, participants recorded where they were, who they were eating with, and whether they were watching television and/or sitting at a table. After each survey day, participants recorded if their intake was representative for that day (or the reason why if it was not), and details of any dietary supplements taken. The survey also contained a series of questions about participants customary eating habits (e.g., the type of milk usually consumed) to facilitate further coding. Food records were returned from the field in real time, to be coded by trained coders who were supervised by dieticians. An ad hoc central server software/database was developed for this purpose, to work in parallel with the codification and verification processes. Food, beverage, and energy and nutrient intakes were calculated from food consumption records using VD-FEN 2.1 software, a Dietary Evaluation Program from the Spanish Nutrition Foundation (FEN), Spain, which was newly developed for the ANIBES study by the FEN and is based mainly on Spanish food composition tables [19], with several expansions and updates. Data obtained from food manufacturers and nutritional information provided on food labels were included. A food photographic atlas was used to assist in assigning gram weights to portion sizes. The VD-FEN 2.1 software was developed to receive information from field tablets every two seconds, and the database was updated every 30 min. Energy distribution and objectives for the Spanish population were used to analyze the overall quality of the diet [16,20].

### 2.3. Evaluation of Misreporting

National diet and nutritional surveys are the most used tools to assess diet, nutrient intake, and the nutritional status of the population. Data collected in the surveys is based on subjects self-reporting. As this method is indirect and has a pseudo-quantitative nature, the surveys frequently report data that do not represent the habitual intake of the studied population and estimates energy intakes (EI) that are not plausible physiologically [21]. In this respect, EFSA has published a protocol that has a harmonized approach to identify misreporting based on a review of the methods used in representative samples of people aged 10–74 years in Europe [22]. EFSA suggests that the data should be reported for the whole population as well as divided into plausible and non-plausible reporters, and so this is included in the present article. EFSA recommendations were followed to calculate misreporting; in which the proposed protocol is based mainly on Goldberg [23] and Black [24]. This method evaluates the reported EI (EIrep) against the presumed energy requirements. EIrep is expressed as a multiple of the mean basal metabolic rate estimate (BMRest), and it is compared with the presumed energy expenditure of the studied population. Then, the ratio EIrep:BMRest is referred to as the physical activity levels (PAL) [22]. 

### 2.4. Statistical Analysis

Values are expressed as median (interquartile range) or as percentage. The non-parametric data were statistically analyzed by the Kruskal–Wallis test. When the Kruskal–Wallis test resulted in differences, multiple comparisons between medians were studied by Mann-Whitney’s U test, while significant differences among groups were allocated by post hoc Student-Newman-Keuls test. Differences were considered significant at *p* < 0.05. To establish if the samples were parametric or non-parametric, the Kolmogorov–Smirnov test was used. Data analysis was performed with SPSS 22.0 software package (IBM Corp., Armonk, NY, USA).

Values obtained for two diagnostic criteria on iron Recommended Dietary Intakes (RDI): iron Dietary Reference Values by EFSA [25] and iron daily Recommended Intakes for the Spanish population as reviewed by Moreiras et al. [12] were used (Table 1). Prevalence of adequacy for iron intakes (% population above 80% RDI) was calculated for each of the RDI and then compared using the McNemar test for paired proportions. The kappa coefficient (ĸ) was used to assess the degree of agreement of the two classification criteria: EFSA and Moreiras et al. Agreement interpretation was based on established categorizations: “poor” (ĸ < 0.000), “slight” (0.000–0.200), “fair” (0.210–0.400), “moderate” (0.410–0.600), “substantial” (0.61–0.80), and “almost perfect” (0.810–1.000).

## 3. Results

A total of 2009 individuals, 996 women and 1013 men, participated in the study. The distribution by age and sex of the sample and the study population were not significantly different from the Spanish population for these age groups. A higher proportion of non-plausible reporters was identified amongst men (53.3%, Table 2). In addition, 75.9% of adults were acknowledged as non-plausible reporters (Table 3). Conversely, a lower proportion of non-plausible reporters was observed in children (5.8%) and adolescents (8.4%).

Median daily iron intakes for the Spanish population aged 9–75 years are shown in Table 4. Males had higher iron intakes than females in the whole sample. Total median iron intakes amongst female were 9.8 mg/day while males were 11.3 mg/day. Significantly higher intake values (*p* < 0.001) were observed in the plausible reporters group in both female and male, with an iron intake of 12.0 mg/day and 14.7 mg/day, respectively.

Prevalence of adequacy for iron intakes (% population above 80% RDI) in the study population is presented by sex and reporting in Table 4 according to the different diagnostic criteria: national (Moreiras et al. [12]) and international (EFSA [25]). The proportion of adequacy for total iron intake in female was 17.0% and 27.3%, and for male it was 57.3% and 77.2% according to the Spanish [12] and EFSA [25] references, respectively (Table 4). The degree of agreement (ĸ) between the two diagnostic criteria, observed in the present study, indicated that it was “moderate” for the prevalence of adequacy between Spanish [12] and EFSA [25] criteria in the case of women (ĸ = 0.526) and “substantial” for men (ĸ = 0.697).

Results of the analysis by age group and reporting are shown in Table 5. Adolescents (11.4 mg/day) and children (11.0 mg/day) had higher total iron intakes than adults (10.4 mg/day) and the elderly (10.2 mg/day). In all age groups, iron intake values in plausible reporters were significantly higher (*p* < 0.001) than in non-plausible reporters. Noteworthy, iron intakes were higher in the adolescent’s plausible reporters group (13.3 mg/day). Conversely, children’s plausible reporters group presented lower iron intake levels (12.2 mg/day). The degree of agreement (ĸ) between the two diagnostic criteria was “fair” for the prevalence of adequacy between Spanish [12] and EFSA [25] criteria in all age groups (0.210–0.400).

When iron intakes were evaluated by geographical distribution, overall, we observed that North Central region (11.4 mg/day) and Northeast (10.9 mg/day) presented higher daily iron intakes, while the Center of the peninsula (9.9 mg/day), Canary Islands (10.1 mg/day) and the South region (10.1 mg/day) had the lowest intakes (Table 6). 

### Contribution of Food and Beverage Groups to Iron Intake

**Figure 2:**
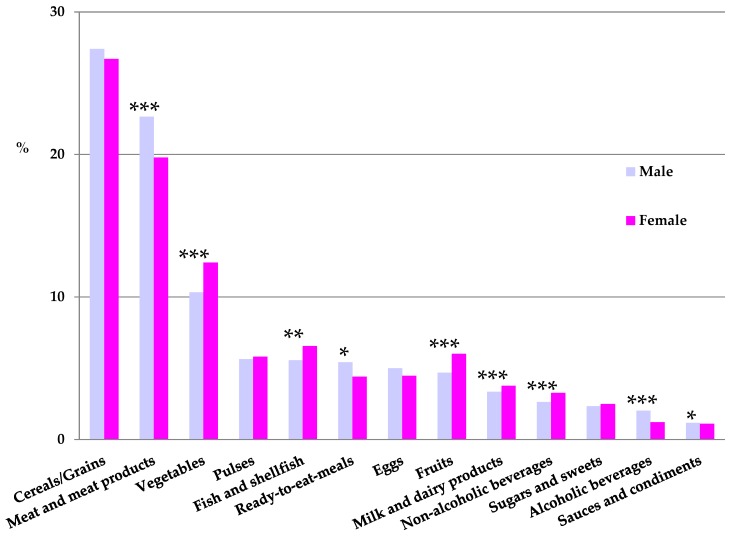
Contribution of food and beverages to iron intake by gender. * *p* < 0.05 difference vs. women (Mann-Whitney’s U test); ** *p* < 0.01 difference vs. women (Mann-Whitney’s U test); *** *p* < 0.001 difference vs. women (Mann-Whitney’s U test).

The contribution (%) of food and beverage categories to the daily iron intake is shown, categorized by genre, in Figure 2. The food groups with the highest mean proportional contribution to total iron intake in both males and females were firstly cereal and grain products (26.7%–27.4%) and meat and meat products (19.8%–22.7%), of which intakes were significantly higher in males (*p* < 0.001). Thirdly, vegetables accounted for a 10.3%–12.4% of iron intakes, being significantly higher (*p* < 0.001) in females. Together, these three food groups contributed to ≥60% of iron intakes of the studied population. 

When analyzing main food group sources for different age groups (Table 7), cereals and grain products were the main sources of iron for the entire sample, especially for adolescents (31.3%–33.1%) and children (30.7%–31.8%), where they were significantly higher than adults and the elderly (*p* < 0.05). Meat and meat products were the second largest contributors, being lowest for the elderly population (17.0%) and significantly highest for children, adolescents, and adult males (23.5%) (Table 7).

Since meat and meat products are the food group with the highest sources of bioavailable heme iron, we decided to further explore the different subgroups and their contribution to iron intakes (Table 8). Total meat contribution was significantly higher (*p* < 0.05) for children (male) (12.9%), adolescent males (13.5%), and adult males (13.3%) vs. the rest of age groups. Red meat provided the same contribution for all age groups. Secondly, sausages and other meat products provided 5.5%–10.2% of total iron intakes, especially in children, adolescents and adult male which were significantly higher (*p* < 0.05) than in adult female and the elderly. On the other hand, the fish and shellfish group contribution was significantly higher (*p* < 0.05) in groups older than 18 years. 

## 4. Discussion

### 4.1. Iron Deficiency and Vulnerable Population Groups

In Europe, iron deficiency is considered to be one of the main nutritional deficiency disorders affecting large fractions of the population, particularly groups such as children and fertile or pregnant women. Moreover, adolescents consuming low energy diets, vegetarians, and vegans are at high risk of iron deficiency [26]. Children and adolescents are consistently considered a group at risk for nutritional deficiencies as their needs increase due to high growth requirements, in addition to some deleterious dietary habits (high consumption of empty calories, “meal skipping”, etc.) [27]. Median total iron intake in children and adolescents from our study was 11 and 11.4 mg/day respectively. Even significantly higher values were observed for plausible reporters and adequacy prevalence (% higher than 80% RDI) was 40.9% and 15.2% for children and adolescents respectively, according to national recommendations. Results from the EnKID Study [28,29], back in 1998 observed that mean daily iron intakes were 14.4 ± 3.1 mg/day and 11.8 ± 2.1 mg/day in boys and girls, respectively. In this population-based, cross-sectional study, that evaluated the dietary habits and nutritional status of Spanish schoolchildren and adolescents aged 2–24 year (*n* = 3534), researchers used 24 h recalls and a food frequency questionnaire. Food patterns of this population group revealed moderate milk consumption, high consumption of dairy products and meat intake, and low consumption of fish, fruit, and vegetables. Noteworthy, in this study, under-reporters were excluded from the analysis [27].

Women of childbearing age are another vulnerable population for iron deficiency for the reasons already discussed. Overall, our results showed that women had a median total iron intake of 9.8 mg/day, with a significantly higher intake of 12.0 mg/day within plausible reporters. Adequacy prevalence as a percentage of population above 80% of national [12] and EFSA [25] recommendations was 17.0% and 27.3% respectively, while in the case of men adequacy increased to 57.0% and 77.2% respectively. The large difference between men and women regarding prevalence of adequate intake calls for attention. Women are at high risk of iron deficiency and should be the ones to increase their iron intake. However, women’s RDI for iron are more difficult to reach (18 mg/day compared to 8 mg/day for men and postmenopausal women). In view of these results, nutritional policies should be created for the female population, from different public administrations, to encourage the consumption of iron-rich foods, as well as to establish nutritional education programs to teach how to promote the ingestion of iron when ingested with foods with less bioavailable iron content. Quintas et al. studied a group of Spanish women (19–35 years, *n* = 130), showing a high incidence of iron deficiency at blood level (10.7%) and a low iron intake (11.1 ± 3.0 mg/day) [30]. Iron intakes and status were also assessed in the Spanish National Survey of Dietary Intake (Encuesta Nacional de Ingesta Dietética España, ENIDE) [9]. Their findings reveal that men’s average daily iron intakes were higher than women’s (15 mg/day vs. 12–14 mg/day) with a high percentage of fertile women that did not achieve recommended intakes. Median intake values were 14–15 mg/day in women and 16 mg/day in men, so that according to EAR, iron intakes were adequate.

### 4.2. Misreporting

The purpose of this pioneer study was to evaluate dietary intakes of iron in the Spanish population according to age, gender, and misreporting, and to examine the contribution from different foods to this mineral’s total intake. To our knowledge, this is the first representative Spanish study considering plausible and non-plausible reporters for assessing dietary iron intake. Understanding the error in self-reported data may help both improving data collection and analyzing the relationships between dietary intake and health outcomes [31]. Maintaining or excluding subjects identified as under-reporters in the analyses is still a matter of debate. When the main issue is to describe eating patterns in a national representative sample, for example, excluding under-reporters is likely to induce a selection bias [32]. The latter may be of special importance when considering micronutrients intakes.

According to the literature, women are more likely to under-report than men, and under-reporting is more common among overweight and obese individuals [10,29]. Other associated characteristics, for which there is less consistent evidence, include age, smoking habits, level of education, social class, physical activity, and dietary restraint. [33]. Our results show that men represent the higher proportion of non-plausible reporters (53.3%). Also remarkably, adults were identified as the major age group who were non-plausible reporters (75.9%), while other studies find that children and adolescents represent higher percentages of misreporting [33].

Low-energy reporters are characterized by a relatively less favorable profile, in terms of education, weight status, sedentary behavior, and eating behaviors. People with low educational background and thus low knowledge on nutrition and health are likely to be both less interested and less compliant in recording food intake [34]. A number of authors have used the Goldberg cut-off to identify “low energy reporters” and to explore their characteristics or to examine the effects of low energy reporting on the data and the conclusions to be drawn from it [9].

Macdiarmid et al. [33] reviewed the main causes of misreporting and stated that the prevalence of under-reporting in large nutritional surveys could range from 18 to 54% of the whole sample, but can be as high as 70% in particular subgroups. Authors declare that the most consistent differences are between men and women and between groups differing in body mass index [33]. Although less likely to occur, the possibility of over-reporting cannot be overlooked. Foods which have a positive *health image* may be over-reported to describe a healthy diet [11].

### 4.3. Iron Food Sources

Detailed information on dietary iron sources is essential to better understand the strengths and weaknesses of the Spanish diet quality and to identify vulnerable population groups. Our results show that higher percentages of iron are provided by cereal and grain products (26.7%–27.4%) throughout all the studied population. This could indicate that the higher iron proportion comes from non-haem iron dietary sources, thus its bioavailability may be compromised. Meat and meat products are the second contributing group (19.8%–22.7%). Results of the ANIBES study regarding main energy sources of the diet showed that the contribution of meat and meat products was categorized as “very high” amongst all age groups [16]. A number of recommendations by public health authorities designate that meat and processed meat products intake should be limited [35]. But, in this regard, as they represent the best haem iron dietary source, a compromise should be reached. According to Hercberg et al., haem iron in haemoglobin and myoglobin in meat, poultry, and fish usually constitutes only 10% or less of the total iron intake in European mixed diets, but the average absorption of haem iron is usually around 25% (but may vary from about 10% to 40%) [26]. The authors find that non-haem iron in cereals, vegetables, fruits, roots, pulses and beans constitutes the main part of dietary iron, although its bioavailability is low (1% ± 5%). Pulses and legumes are minor as iron sources amongst the studied population (4.5%–7.6%), being lower in children and higher in the elder group. Results obtained in the ENIDE dietary survey in Spain [36] showed that the higher percentage of iron contribution was from legumes and seeds (23%), with fish and shellfish and meat and meat products being second (19%) and third (16%), respectively. Noteworthy, cereal and grain products were the fourth iron source, accounting for 11% of total intakes [36]. It is important to acknowledge that there are a number of factors that affect iron absorption into the gastrointestinal system and thus iron bioavailability: calcium, phytates in cereals and legumes, and phenolic compounds found in tea, coffee, and other beverages bind iron and restrict its availability for absorption, while meat and vitamin C found in fruit and vegetables enhance the potential availability of iron for mucosal uptake [11]. The fact that between 6.7% and 14.7% of total dietary iron comes from the vegetable group indicates that vitamin C from this sources is potentially also consumed, although more information is required on iron absorption enhancers and inhibitors. 

Mandatory iron fortification of wheat flour is implemented in a number of European countries such as the UK [26], where it accounts for 6%–10% of dietary intakes; but at present, only a voluntary food fortification scheme takes place in Spain, where food products such as fortified breakfast cereals and bars are available. It is noteworthy this type of products were not assessed in our survey.

The strengths of this study include the careful design, protocol, and methodology used in the ANIBES study, conducted among a random representative sample of the Spanish population aged 9–75 years. Food consumption assessment was performed using digital tablets and included a thorough quality control process. Main limitations are the cross-sectional design and the use of newly developed technologies for some age groups (i.e., the elderly).

The existence of potential iron insufficiency may be related to the rapid evolution of the dietary model and lifestyle modifications over the last few decades in our country. Indeed, in the last several generations, a reduction in total calorie intake was observed (due in part to a reduction in physical activity) [16] which has possibly led to a decrease in iron intake along with that of most other dietary micronutrients. Moreover, the increase in consumption of foods containing “empty calories,” lacking or being low in trace elements or vitamins, has contributed to a decrease in the micronutrient density in diet.

## 5. Conclusions

In conclusion, the iron intakes from the ANIBES study population in Spain were studied and assessed, including plausible and non-plausible reporters. Total median daily iron intake levels observed were low for women and for men. Significantly higher iron intake values were observed among plausible reporters from both sexes and all age groups. The major proportion of dietary iron sources were cereal and grain products, which could indicate that the proportion of non-heme iron intake is higher in our study population.

Our results shows that the prevalence of adequacy for iron by the EFSA [25] criteria was higher than the one from the national standard (Moreiras et al. [12]). Therefore, there is a need to define standard dietary reference intake to determine inadequate mineral intakes in the Spanish population.

Finally, to optimize iron status, it is still desirable to encourage a varied diet with adequate attention to sources of haem iron, and more emphasis should be given to the enhancing or inhibitory factors influencing non-haem iron absorption by means of adequate recommendations regarding dietary habits.

## Figures and Tables

**Table 1:** Recommended dietary intakes (RDI) by Moreiras and EFSA.

Age Group (Years)	Moreiras et al. [12]	EFSA [25]
	Fe (mg/Day)	Fe (mg/Day)
Men		
9	9	11
10	12	11
11	12	11
12	12	11
13–17	15	11
18–19	15	11
20–49	10	11
50–59	10	11
≥60	10	11
Women		
9	9	11
10	18	11
11	18	11
12	18	13
13–17	18	13
18–19	18	16
20–49	18	16 ^a^
50–59	10	16 ^a^
≥60	10	11

^a^ For postmenopausal women, Daily Recommended Iron Intake (DRI) is the same as for women ≥60 years.

**Table 2:** Description of the ANIBES sample by gender and reporting.

Gender	Reporting	
	Plausible Reporters	Non-Plausible Reporters
Female	57.3% (*n* = 311)	46.7% (*n* = 685)
Male	42.7% (*n* = 232)	53.3% (*n* = 781)

**Table 3:** Description of the ANIBES sample by gender reporting and age group.

		Children	Adolescents	Adults	Elderly
Gender	Female	7.7%	6.6%	76.2%	9.5%
		(*n* = 87)	(*n* = 74)	(*n* = 857)	(*n* = 107)
	Male	10.9%	11.8%	68.8%	8.5%
		(*n* = 126)	(*n* = 137)	(*n* = 137)	(*n* = 137)
Reporting	Plausible reporters	17.8%	11.3%	64.2%	6.7%
		(*n* = 120)	(*n* = 76)	(*n* = 433)	(*n* = 45)
	Non-plausible reporters	5.8%	8.4%	75.9%	10.0%
		(*n* = 93)	(*n* = 135)	(*n* = 1222)	(*n* = 161)

**Table 4:** Iron intake (mg/day) and prevalence of adequate (% population above 80% RDI) in ANIBES sample by gender and reporting and agreement (ĸ) between Spanish [12] and EFSA references [25].

Gender	Iron (mg/Day)	% Above 80% RDI Moreiras (Spain)	% Above 80% RDI EFSA	RDI Agreement (Kappa) Moreiras vs. EFSA
Women				
Total *n* = 996	9.8 (7.9–11.9)	17.0	27.3 ^##^	0.526
Plausible *n* = 331	12.0 *** (10.3–13.8)	24.8	50.5 ^###^	0.570
Non-plausible *n* = 685	8.8 (7.3–10.6)	13.4	19.8 ^###^	0.462
Men				
Total *n* = 1013	11.3 (9.0–14.0)	57.3	77.2	0.697
Plausible *n* = 232	14.7 *** (12.4–17.1)	84.0	100.0 ^##^	-
Non-plausible *n* = 781	10.3 (8.4–12.7)	49.3	70.4 ^##^	0.694

Values are median (interquartile range) per group. *** *p* < 0.001 difference plausible vs. non-plausible (Mann-Whitney’S U test). ^##^ *p* < 0.01 differences between Moreiras et al. and EFSA references (McNemar test). ^###^ *p* < 0.001 differences between Moreiras et al. and EFSA references (McNemar test).

**Table 5:** Iron intake (mg/day) and prevalence of adequate (% population above 80% RDI) in ANIBES sample by age group and reporting and agreement (ĸ) between Spanish [12] and EFSA [25] references.

Age Group	Iron (mg/Day)	% Above 80% RDI Moreiras (Spain)	% Above 80% RDI EFSA	RDI Agreement (Kappa) Moreiras vs. EFSA
Children				
Total *n* = 213	11.0 (9.2–12.8)	40.9	77.9 ^###^	0.345
Plausible *n* = 120	12.2 *** (10.4–14.0)	54.2	94.2 ^###^	0.255
Non-plausible *n* = 93	9.2 (8.0–11.1)	23.7	57.0 ^##^	0.540
Adolescents				
Total *n* = 211	11.4 (9.1–13.4)	15.2	73.0 ^###^	0.243
Plausible *n* = 76	13.3 *** (11.6–15.4)	27.6	90.8 ^###^	0.212
Non-plausible *n* = 135	10.0 (8.1–11.8)	8.2	63.0 ^###^	0.277
Adults				
Total *n* = 1655	10.4 (8.4–12.9)	36.9	47.9 ^###^	0.233
Plausible *n* = 433	13.0 *** (11.0–15.6)	47.8	63.3 ^##^	0.730
Non-plausible *n* = 1222	9.6 (7.8–11.8)	33.0	42.5 ^###^	0.709
Elderly				
Total *n* = 206	10.2 (7.9–12.6)	52.9	68.0 ^###^	0.170
Plausible *n* = 45	12.7 *** (10.9–17.2)	88.9	100.0 ^###^	-
Non-plausible *n* = 161	9.5 (7.5–11.5)	42.9	59.0 ^###^	0.842

Values are median (interquartile range) per group. *** *p* < 0.001 difference No misreporting vs. Misreporting (Mann-Whitney’s U test); ^##^ *p* < 0.01 differences between Moreiras and EFSA references (McNemar test); ^###^ *p* < 0.001 differences between Moreiras and EFSA references (McNemar test).

**Table 6:** Iron intake (mg/day) by geographical distribution.

Geographical Distribution (Nielsen Areas)	Iron (mg/Day)
Barcelona (Metropolitan Area)	10.8 (8.8–13.1)
Canary Islands	10.1 (7.8–13.3)
Center	9.9 (8.2–13.2)
East	10.6 (8.3–13.2)
Madrid (Metropolitan Area)	10.2 * (8.1–12.7)
Northeast	10.9 (8.7–13.4)
Northwest	10.6 (8.6–12.7)
North Central	11.4 (9.6–14.0)
South	10.1 ** (8.2–12.4)

Values are median (interquartile range per group); * *p* < 0.05 difference vs. North Central (Bonferroni test); ** *p* < 0.01 difference vs. North Central (Mann-Whitney’s U test).

**Table 7:** Contribution of food and beverages to iron intake by gender and age group.

	Children		Adolescents		Adults		Elderly	
	Male	Female	Male	Female	Male	Female	Male	Female
Cereals/Grains (%)	30.7 ^a^	31.8 ^a^	33.1 ^a^	31.3 ^a^	26.9 ^b^	26.9 ^b^	24.0 ^c^	24.6 ^b,c^
Meat and meat products (%)	23.1 ^d^	20.1 ^d,e^	23.5 ^d^	20.3 ^d,e^	22.9 ^d^	20.1 ^e^	18.0 ^e^	17.0 ^e^
Vegetables (%)	7.1 ^f^	7.9 ^f^	6.7 ^f^	7.7 ^f^	10.8 ^g^	12.4 ^h^	13.0 ^h,i^	14.7 ^i^
Pulses (%)	4.5	6.4	4.9	5.7	5.6	5.8	7.6	6.0
Fish and shellfish (%)	3.8 ^j^	4.0 ^j^	3.7 ^j^	4.3 ^j^	5.7 ^k^	6.5 ^l^	6.9 ^l,m^	7.9 ^m^
Ready-to-eat-meals (%)	6.6 ^n^	5.7 ^n,o,p^	7.6 ^n,o^	6.2 ^n^	5.3 ^o,p^	4.6 ^p^	2.9 ^p^	2.1 ^q^
Eggs (%)	4.6	4.3	5.0	4.7	5.0	4.4	5.5	5.3
Fruits (%)	3.3 ^r^	3.8 ^r^	2.4 ^s^	3.8 ^r^	4.6 ^r^	5.7 ^t^	8.4 ^u^	10.2 ^w^
Milk and dairy products (%)	4.7 ^x^	4.3 ^y^	3.8 ^y^	4.0	3.3 ^y^	3.7 ^z^	2.8 ^z^	3.9 ^y^
Non-alcoholic beverages (%)	2.5 ^α^	2.6 ^α,β^	2.0 ^α^	2.6 ^α^	2.8 ^β^	3.2 ^γ^	2.9 ^β,γ^	3.4 ^δ^
Sugars and sweets (%)	6.1 ^ε^	5.8 ^ε,ζ^	4.7 ^ζ^	6.5 ^ε,ζ^	2.0 ^η^	2.4 ^θ^	1.2 ^ι^	0.7 ^ι^
Alcoholic beverages (%)	-	-	-	-	2.1 ^κ^	1.3 ^λ^	4.8 ^μ^	2.0 ^λ^
Sauces and condiments (%)	1.0 ^ν^	1.6 ^ν^	1.1 ^ν^	1.3 ^ν^	1.2 ^ν^	1.1 ^ν^	0.7 ^ξ^	0.8 ^π^

Values are percentage. All differences are *p* < 0.05 (Student-Newman-Keuls test). Different superscript lowercase letters indicate statistical significance in each row.

**Table 8:** Contribution of main meat and fish types to iron intake by gender and age group.

	Children		Adolescents		Adults		Elderly	
	Male	Female	Male	Female	Male	Female	Male	Female
Meat (total) (%)	12.9 ^a,b^	9.8 ^a^	13.5 ^b^	10.7 ^a^	13.3 ^b^	12.1 ^a^	10.6 ^a^	10.9 ^a^
Red meat (%)	5.3	5.1	5.4	2.8	5.5	4.8	5.1	4.8
White meat (%)	2.9 ^c,d^	1.7 ^e^	3.3 ^c,d^	3.1 ^c,d^	3.3 ^d^	3.0 ^c,d^	2.4 ^c,d,e^	2.1 ^c,e^
Poultry (%)	4.7 ^f^	3.0 ^g^	4.8 ^f^	4.9 ^f,g^	4.5 ^f^	4.3 ^f,g^	3.1 ^f,g^	4.0 ^f,g^
Viscera and offal (%)	0.0 ^h,i^	0.3 ^h,i^	0.0 ^h^	0.1 ^h,i^	0.6 ^i^	0.4 ^h,i^	1.0 ^j^	0.6 ^h,i^
Sausages and other meat products (%)	10.2 ^k^	10.0 ^k^	10.0 ^k^	9.5 ^k^	9.0 ^k^	7.5 ^l^	6.4 ^l,m^	5.5 ^m^
Fish and shellfish (total) (%)	3.8 ^n^	4.0 ^n^	3.7 ^n^	4.3 ^n^	5.7 ^o^	6.5 ^p^	6.9 ^p,q^	7.9 ^q^
White fish (%)	1.1 ^r,s,t^	1.6 ^s,t,u^	0.7 ^r^	0.8 ^r^	1.1 ^r^	1.2 ^r,s^	2.0 ^t,u^	2.5 ^u^
Blue Fish (%)	0.5 ^v,w^	0.7 ^v,w^	0.3 ^v^	1.0 ^v,w^	0.8 ^w^	1.0 ^w^	1.6 ^y^	0.9 ^w^
Shellfish (%)	1.6 ^z,α^	1.1 ^z^	1.7 ^z^	1.5 ^z^	2.2 ^α^	2.6 ^α^	2.2 ^α^	2.7 ^z,α^
Canned Fish (%)	0.6 ^β^	0.6 ^β^	1.0 ^β^	0.9 ^β^	1.7 ^γ^	1.7 ^γ^	1.0 ^β,γ^	1.8 ^γ^

Values are percentage. All differences are *p* < 0.05 (Student-Newman-Keuls test). Different superscript lowercase letters indicate statistical significance in each row.
